# Homotopy-Theoretic Study & Atomic-Scale Observation of Vortex Domains in Hexagonal Manganites

**DOI:** 10.1038/srep28047

**Published:** 2016-06-21

**Authors:** Jun Li, Fu-Kuo Chiang, Zhen Chen, Chao Ma, Ming-Wen Chu, Cheng-Hsuan Chen, Huanfang Tian, Huaixin Yang, Jianqi Li

**Affiliations:** 1 Beijing National Laboratory for Condensed Matter Physics, Institute of Physics, Chinese Academy of Sciences, Beijing 100190, China; 2 University of Chinese Academy of Sciences, Beijing 100049, China; 3 National Institute of Clean-and-Low-Carbon Energy, Beijing 100209, China; 4 Center for Condensed Matter Sciences, National Taiwan University, Taipei 106, Taiwan; 5 Department of Physics and Astronomy, Rutgers University, Piscataway, New Jersey 08854, USA; 6 Department of Physics and State Key Laboratory of Low-Dimensional Quantum Physics, Tsinghua University, Beijing 100084, China

## Abstract

Essential structural properties of the non-trivial “string-wall-bounded” topological defects in hexagonal manganites are studied through homotopy group theory and spherical aberration-corrected scanning transmission electron microscopy. The appearance of a “string-wall-bounded” configuration in *R*MnO_3_ is shown to be strongly linked with the transformation of the degeneracy space. The defect core regions (~50 Å) mainly adopt the continuous *U*(1) symmetry of the high-temperature phase, which is essential for the formation and proliferation of vortices. Direct visualization of vortex strings at atomic scale provides insight into the mechanisms and macro-behavior of topological defects in crystalline materials.

In recent decades, the structural features of topological defects that are expected to arise from phase transitions and, in particular, after spontaneous symmetry breaking, have been extensively studied in particle physics and condensed matter physics^1^,^2^,^3^,^4^,^5^. Investigation of related stable configurations, such as domain walls, strings, monopoles, textures and other ‘hybrid’ entities helps widen our vision of the cosmos and unveils many hitherto unknown physical properties of crystalline materials that arise from the striking geometric patterns of their order parameter fields^6^,^7^,^8^,^9^,^10^,^11^. In describing the essential features of topological defects mathematically, the elements of homotopy groups associated with the symmetry of the order parameter space are used to classify these defects, and the exact sequences of homotopy groups can be used to examine defect transformations associated with alterations of degeneracy space^4^,^5^,^12^,^13^,^14^.

The sixfold vortex domains observed in hexagonal manganites *R*MnO_3_ (*R* = Ho-Lu, Y, and Sc) are regarded to be a typical type of topological defect and are correlated with many intriguing physical phenomena^15^,^16^,^17^,^18^,^19^,^20^,^21^. In YMnO_3_, the crystal adopts centrosymmetric *P*6_3_/*mmc* (D_6*h*_, *Z* = 2) structure at temperature above the structural phase transition (*T*_s_ ≈ 1270 K), and then symmetry decreases to *P*6_3_*cm* (C_6*v*_, *Z *= 6) below *T*_*s*_, a transformation induced mainly by the condensation of the *K*_3_ non-polar mode. This transition leads to a trimerized tilting of the 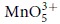 bipyramids and to corrugation of intercalated Y^3+^ layers. Six distinct azimuthal angles (φ) of tilt, separated by 60°, result in six different configurations of unit cells (labeled by α^+^, β^−^, γ^+^, α^−^, β^+^ and γ^−^). These six structures are arranged in sequence (clockwise or anti-clockwise) around a center, and they are partitioned by six domain walls^22^,^23^,^24^,^25^,^26^. Because of its substantial effect on local physical properties, the domain wall microstructure has been extensively studied via atomic-resolved transmission electron microscopy (TEM) and first-principles calculation. Indeed, the observed structural properties are quite interesting, and there is ongoing debate about the best structural model for the domain walls, and for the vortex core as well^27^,^28^,^29^,^30^,^31^,^32^.

In this paper, we report our detailed theoretic and experimental study of the internal structure of two types of topological defects: domain walls and vortex cores. The temperature dependent transformation of defects and the variations of the order parameters inside the topological defects are investigated by homotopy theory from the viewpoint of topology and by numerical simulations from the viewpoint of free energy. In addition, atomic image analyses (considering “overlapping effect”) have been carried out to reveal specific structural features within the defects.

## Results

### Degeneracy space

**Figure 1:**
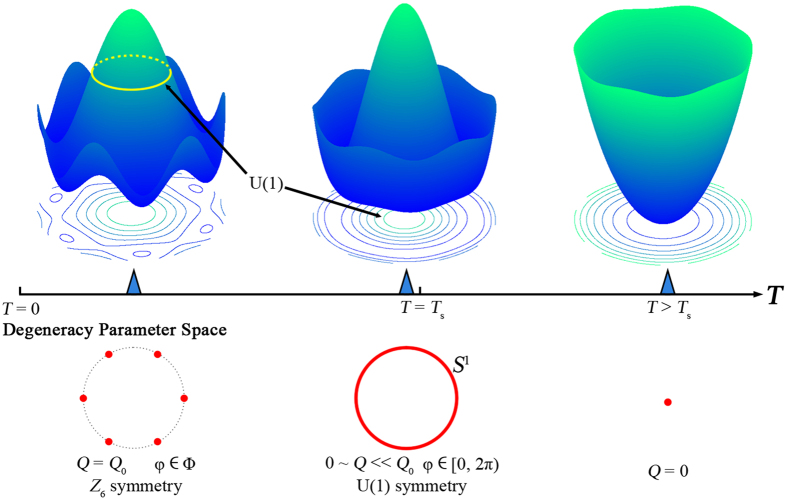
Variation of potential**-**energy surface and degeneracy space with temperature. (**a**) At low temperature, the surface can be represented as a “Mexican-hat” structure with six wells in the brim; the degeneracy space is composed of six distinct points. (**b**) As temperature approaches *T*_s_, the barriers between adjacent wells disappear gradually and the degeneracy space becomes a circle. (**c**) At *T* > *T*_s_, the minimum of potential-energy appears at *Q *= 0 and the degeneracy space shrinks into a single point.

In order to investigate the topological properties of defects, the media are characterized by order parameter fields valued in a space of degeneracy^1^,^2^,^3^,^4^,^5^. In *R*MnO_3_, the amplitude (*Q*) that reflects the relative strength of the *K*_3_ mode and the azimuthal tilt angle (φ) of the 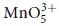 bipyramid is chosen as two components of the primary order parameter, and the amplitude of the 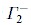 mode (*P*) is the secondary order parameter^24^,^26^. The degeneracy space can be determined by minimization of the temperature-dependent bulk free energy density. Figure 1(a–c) show the temperature dependent variation of potential-energy surface. At low temperatures, the surface adopts the form of a “Mexican hat” with six wells, and the energetic barriers between different trimerized states become negligible with increasing *T*. Next, the minimization of bulk free energy decreases *Q* remarkably and give rise to continuous variation of φ (from 0 to 2π), and then the degeneracy space of φ transforms from ***R***_**1**_ (composed of six discrete points) with *Z*_6_ symmetry to ***R***_**2**_ (with the form of a unit circle *S*^1^) with *U*(1) symmetry^33^. When *T* > *T*_s_, the minimum is at *Q* = 0, suggesting that the system transforms to a centro-symmetric phase without distortion, and the degeneracy space ***R***_**3**_ shrinks into a single point, as shown in supplementary section S1.

### Homotopy theory for *R*MnO_3_

In homotopy theory, topological defects are classified by the elements of homotopy groups associated with the symmetry of the order parameter space. For *R*MnO_3_, space ***R***_**1**_ is composed of six discrete points, so the zeroth homotopy group π_0_(***R***_**1**_, *x*_0_) (*x*_0_: the base point in order parameter space) should be nontrivial and has a one-to-one correspondence with the set of connected components of ***R***_**1**_. Therefore the elements of π_0_(***R***_**1**_, *x*_0_) classify domain walls. Meanwhile, the fundamental homotopy group π_1_(***R***_**1**_, *x*_0_) and any higher order homotopy groups are all trivial because of the discrete *Z*_6_ topology of ***R***_**1**_. Assumption the degeneracy space of a system remains at ***R***_**1**_, even when temperature varies, domain walls are the only possible topological defects (see supplementary section S2, Fig. S2(a–c), and Movie I). When temperature approaches *T*_s_, the degeneracy space expands to ***R***_**2**_, which is isomorphic to a unit circle *S*^1^. In such a condition, only the fundamental homotopy group is non-trivial, and it is isomorphic to the additive group of integers, i.e. π_1_(***R***_**2**_, *x*_0_) = *Z* (*Z* = {0, ±1, ±2, …}). Similarly, this suggests that only one-dimensional vortex strings are the only preferred type of topological defect if the degeneracy space is unchanged. Above the phase transition temperature *T*_s_, the degeneracy space shrinks, resulting in trivial homotopy groups, and no topological defects exist.

However, the vortex structure observed in *R*MnO_3_ at room temperature is not constructed of domain walls alone, nor of strings alone, but of a combination of walls and strings. This implies that, with decreasing temperature, the topological defects must transform, accompanied by alternation of the degeneracy space. An exact homotopy sequence can describe this process^14^. Because ***R***_**1**_ is a subset of ***R***_**2**_, the exact sequence is:


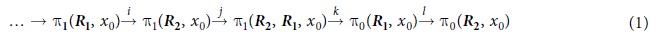


where π_1_(***R***_**1**_, *x*_0_) = 0 (the trivial group), π_1_(***R***_**2**_, *x*_0_) = *Z*, π_0_(***R***_**1**_, *x*_0_) = *Z*_6_, and π_0_(***R***_**2**_, *x*_0_) = 0. Homomorphism *i* maps π_1_(***R***_**1**_, *x*_0_) to the identity element of π_1_(***R***_**2**_, *x*_0_), so none of the non-trivial elements in π_1_(***R***_**1**_, *x*_0_) are images of *i*, nor are the kernels of *j*; instead, they are mapped onto the non-trivial elements in π_1_(***R***_**2**_, ***R***_**1**_, *x*_0_). According to the theorem, the kernel ker(*k*) of homomorphism *k* is a normal subgroup of π_1_(***R***_**2**_, ***R***_**1**_, *x*_0_), and the quotient group π_1_(***R***_**2**_, ***R***_**1**_, *x*_0_)/ker (*k*) is isomorphic to the image im(*k*) of *k*. Because ker (*k*) = im (*j*), and im (*k*) = ker (*l*), we have


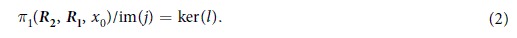


Similarly,


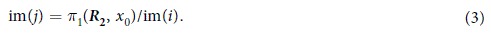


By combining these two equations, we obtain π_1_(***R***_**2**_, ***R***_**1**_, *x*_0_) = *Z* × *Z*_6_, which implies that, although a representative loop in real space around a string may start and end in ***R***_**1**_, the loop necessarily traverses a region (or regions) of ***R***_**2**_ as it encircles the string. Normally, this region will be narrow for energetic reasons and form walls terminating on the vortex string. The elements (*m*, *n*) in π_1_(***R***_**2**_, ***R***_**1**_, *x*_0_) suggest that pure domain walls and “string-wall-bounded” vortex structures are permitted forms of topological defects. The vortices can be classified by the elements (*m*, *n*) with *m *≠ 0, and elements (0, *n*) with *n *≠ 0 can classify the isolated domain walls that are not bounded with strings, i.e. the stripe, circle or annular domains that can coexist with vortices^34^,^35^ (see supplementary section S2). Normally, vortices with a low winding number *m *= ±1 (i.e. the vortex and anti-vortex) are the most common structures observed in *R*MnO_3_. On the other hand, when *T* > *T*_s_ and the degeneracy space shrinks into ***R***_**3**_, all vortex cores become nonsingular in association the annihilation of strings. This transition corresponds to a process in which all elements in π_1_(***R***_**2**_, *x*_0_) are mapped onto the identity (π_1_(***R***_**3**_, *x*_0_) = 0) by the following homomorphism *m*:


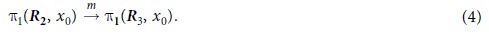


This fact suggests that the bounded structure of strings and walls does not appear immediately after the structural phase transition. Instead, at first, only vortex strings emerge from the topological defect-free, high temperature phase. With declining temperature, domain walls appear progressively in string-attached or isolated forms, whereupon ***R***_**1**_ becomes dominant. Since the intrinsic topology of strings is not broken by alternation of the degeneracy space in this system, it is protected by the formation of “string-wall-bounded” structures and high temperature residual features within vortex cores and domain walls. Thus the cores and walls appear in distinct regions that are composed mainly of the order parameter values belonging to ***R***_**2**_.

### Numerical simulation

**Figure 2:**
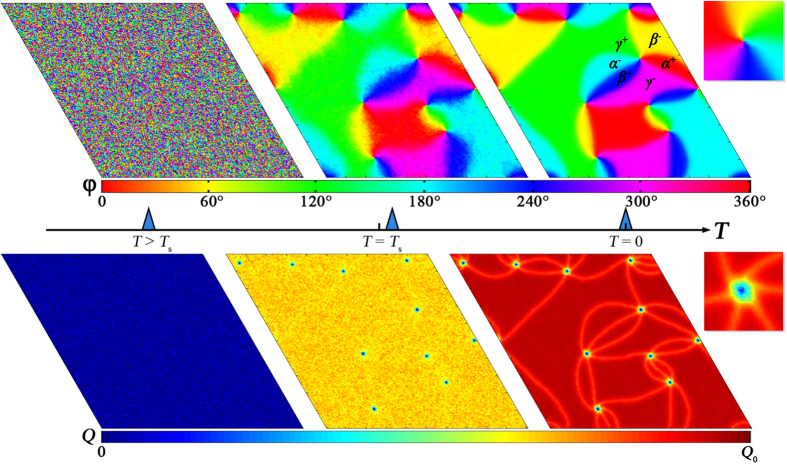
Simulation of the order parameter fields on a 200 × 200 rhombus lattice. (**a**–**c**) Variation of φ (upper panel) and *Q* (lower panel) field with decreasing temperature. Vortex cores appear just below the high temperature structural transition and bounded domain walls appear at lower temperature. A typical core region is enlarged in the right boxes.

Evolution of order parameter field with changing temperature, described in the previous section, is depicted in Fig. 2(a–c), which were obtained from numerical simulation (see supplementary section S3). These three images correspond to three typical states at *T* > *T*_s_, *T*_s_ > *T* ≫ 0 and *T *= 0, respectively. Figure 2(a) shows a defect-free homogenous state with all *Q* values in close proximity to 0. Just below the structure phase transition temperature *T*_s_, *Q* increases slightly to minimize bulk free energy, which remains insensitive to the value of φ, so there is no preference for φ at this stage. However, the spatially configuration of order parameter field has noticeable impact on the gradient energy, so smooth variation of φ among adjacent sites is energetically preferred. The system in this state is analogous to the quasi-liquid phase of the *x*-*y* model in spin system because their degeneracy spaces both adopt *U*(1) symmetry, accompanied by the topological excitations (bounded vortex-antivortex pairs without domain walls) that minimize the total energy of the system^36^, as shown in Fig. 2(b). A further decrease of temperature results in the increase of *Q*, which drives the system into six-fold degeneracy; then domains/domain walls emerge, lowering both the local bulk energy and gradient energy, and each core is surrounded by six domain walls, as shown in Fig. 2(c). It is also notable that the positions of vortex cores change little from Fig. 2(b,c) and that no nucleation or annihilate of cores is observed. This implies that these strings are stable across a large temperature region. The density of vortex cores is controlled mainly by the rate at which the temperature decreases across *T*_s_. During the transformation from Fig. 2(b,c), the intrinsic topology of strings is not affected by symmetry-breaking of the degeneracy space, and it is protected by the formation of “string-wall-bounded” structures. So order parameter values that belong to ***R***_**2**_ can be preserved within vortex cores and walls, as shown in the two enlarged panels in Fig. 2. With such microstructure features, the high stability of vortex cores under external electric field is also demonstrated by our simulations, which coincide with our experimental results (see supplementary Figs S3 and S4).

In analyzing the evolution of vortex pattern, we find that the emergence of *U*(1) is critical for the formation of vortices, even though the final state is of *Z*_6_ symmetry (see Movie II and III in supplementary materials). Note also that theoretical simulations based on phase-field methods or six-state clock model can yield certain results in good agreement with our conclusion: the initial states set for Monte-Carlo calculations are disordered states that can be excited only at high temperatures, the calculation steps correspond to a quenching or annealing process accompanied by spontaneous symmetry breaking from *U*(1) to *Z*_6_, and the six-fold vortices observed in the final state are the inevitable product of this evolution process^37^,^38^,^39^,^40^,^41^.

### Relationship between order parameter and structural distortion

**Figure 3:**
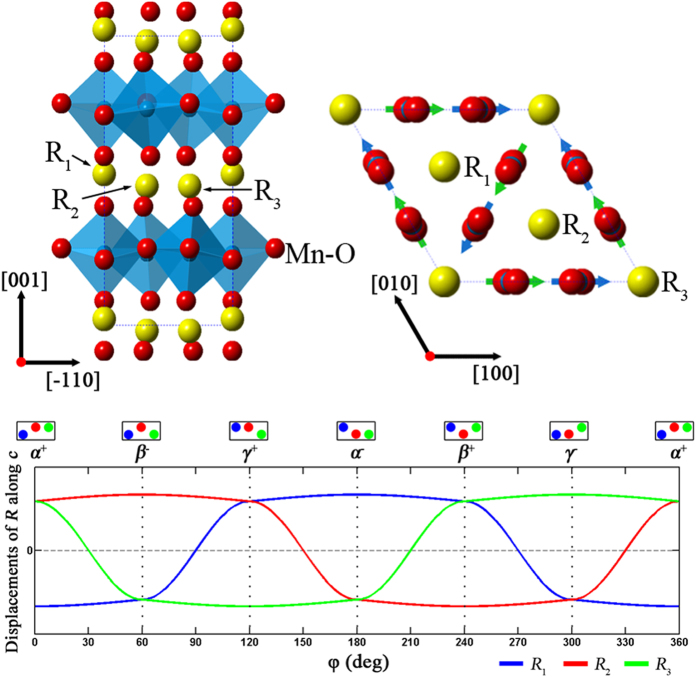
Relationship between order parameter and atomic structure. (**a**) *R*MnO_3_ unit cell with *P*6_3_*cm* symmetry observed along the [110] (left) and [001] (right) directions. When this structure is distorted into 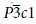 or *P*3*c*1, the buckling state of *R* layers and the tilt direction of 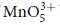 bipyramids change accordingly. (**b**) Relationship between displacements of *R* atoms and parameter φ.

Because the structural distortions are associated with the condensation of the *K*_3_ and 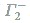 modes, the corrugation state of *R* layers will change in accord with the variation of the order parameters^24^,^26^,^31^,^42^. Group -theoretical analysis shows that there are three distinct symmetry-allowed isotropy subgroups (*P*6_3_*cm*, 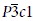 and *P*3*c*1) for the *K*_3_-irreducible representation of *P*6_3_/*mmc*, so these three structures can exist in the *R*MnO_3_ system^42^, and they correspond to φ ∈ Φ, φ ∈ Φ’ and φ ∉ (Φ∪Φ’), respectively (Φ = {0, π/3, 2π/3, π, 4π/3 and 5π/3}, Φ’ = Φ + π/6). Figure 3(a) shows the *P*6_3_*cm* unit cell along the direction in which displacements of *R* atoms are obviously visible. The relationship between displacements of *R* atoms and φ is summarized in Fig. 3(b). (This relationship is qualitatively verified by our first-principles calculation, see supplementary section S4). In addition, because the value of *Q* can be used to quantify the amplitude of the *K*_3_ mode^24^, a decrease of *Q* is accompanied by a reduction of atomic displacements at all *R* sites. Our simulation results show that *Q* decreases only slightly and φ deviates little from Φ within domain walls, so it is reasonable that the unit cells in these regions do not show significant difference from the bulk regions. This coincides with the results given in ref. 31: although φ deviates from Φ within several unit cells, the absolute displacements of *R* atom change little at domain walls. However, more significant deviation of the order parameter from ***R***_**1**_ space happens in the core regions: φ varies continuously around the center, and *Q* decreases dramatically from outside in. So, in contrast to what we observed in the bulk region with *P*6_3_*cm* symmetry, the atomic structure at a vortex core is more likely to adopt the features of the high energy phase 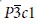 and *P*3*c*1, so significant distortions of *R* layers are expected to be found within the cores.

### Atomic image at domain wall

**Figure 4:**
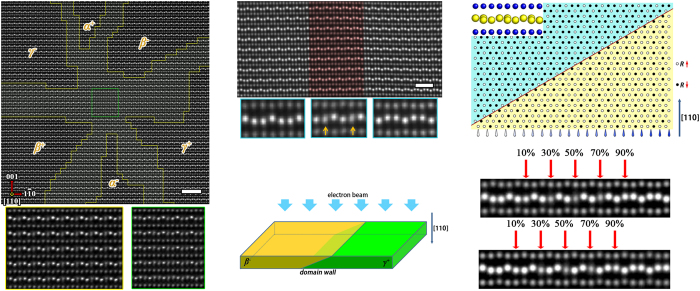
Experimental observation and image simulation of topological defects. (**a**) STEM image of Y_0.9_In_0.1_MnO_3_. Six kinds of domains appear separately around the vortex core. The region in which the contrast of 1/3 Y atoms is ambiguous is delineated by yellow lines and the region in which the contrast of 2/3 Y atoms is ambiguous is indicated by the green box. The typical contrast features of these two regions are shown in the respective enlarged boxes. Scale bar: 2 nm. (**b**) STEM image obtained along the [110] axis in YMnO_3_. The red region separates two domains. Typical contrast features in these three regions are shown below. Scale bar: 1 nm. (**c**) Schematic of typical relative orientation between domain wall and electron beam. (**d**) Distribution of *R* atoms on the ***ab*** plane in a region that contains an inclined domain wall; the hollow circles and solid circles correspond to upward *R* and downward *R*, respectively. A projection along the [110] direction is insert in the top left corner. (**e**) Simulated STEM images based on the structural mode of Fig. 4d. The two kinds of domain walls (upper and lower) show similar contrast changes, as revealed in the experimental images.

In order to clarify the atomic structural features within vortex cores in *R*MnO_3_, we employed high-resolution high angle annular dark field (HAADF) STEM. Figure 4(a) shows an atomically resolved HAADF image of a vortex structure in Y_0.9_In_0.1_MnO_3_, taken along the [110] crystal orientation. The sample thickness in this region is estimated to be 70 nm by electron energy loss spectra. Slight doping of indium in YMnO_3_ can increase the vortex core density, helping one to find proper vortex cores in TEM samples^43^. Six kinds of domains, denoted as α^+^, β^−^, γ^+^, α^−^, β^+^ and γ^−^, with alternating polarization and structural phases, can be identified in this region. However, it is commonly noted that the domain boundaries often show a complex variation of atomic contrast within a few (or tens of) unit cells, where the shift of *R* ions differs from that in domain regions. In vortex center, the imaging of Y columns becomes much more complicated (enlarged image is shown in Fig. S6). This phenomenon implies that unfamiliar structural features may exist within core regions.

An experimental image for a typical domain wall in YMnO_3_ is reproduced in Fig. 4(b). It is clear that the shifts of Y atoms yield a polarization change from left to right. In particular, the atomic structure in the middle region (with a span of about 5 unit cells and marked in red) shows irregular image streaking. Careful examination reveals that one in every three Y atoms become ambiguous, the less bright atomic columns are accompanied by atomic streaking along the ***c*** axis. This structural feature is obvious in the middle region and becomes invisible at both sides of the red-marked region. Experimental observations of a variety of samples show that atomic contrast anomalies always occur on the 4(b) Wyckoff positions in *P*6_3_*cm* symmetry. The “down-down-up” poling configuration can be converted into “down-up-up” or “up-down-up”, depending on whether the transition starts at the (2/3, 1/3, *z*) or (0, 0, *z*) site. Similar phenomena also happen in other kinds of domain walls (See Fig. S7). Based on theoretical simulations and experimental observations, we suggest that the contrast anomalies observed at domain walls arise from the spatial structure of these defects (see supplementary section S6): in TEM experiments, we cannot expect that domain walls are always parallel to the observation direction (e.g. the [110] zone axis in the present case). Considering a slice sample perpendicular to the [110] direction, as shown in Fig. 4(c), two adjacent domains partially overlap along [110] because of the sloped domain wall. Figure 4(d) is a schematic atomic model illustrating a profile projection of the slice (only *R* atoms are shown). Along the [110] direction, some columns contain oppositely distorted *R* atoms (the inset of Fig. 4(d)). These columns are arranged periodically, and the ratio between atoms with opposite distortion varies with the depth of the domain wall. Since this kind of structural feature could affect the channeling effect and dynamic scattering process when electrons pass through a sample^44^,^45^, the contrast of these atomic columns will change significantly in comparison with bulk regions. In order to verify the influence of this “overlapping effect” on HAADF imaging at domain walls, we performed STEM simulations via the QSTEM software package^46^. These calculations are based on the “sharp transition model” in which ferroelectric polarization flips immediately at the wall^31^. The result is shown in Fig. 4(e). As the proportion of upward shifted Y atoms increases from 10% to 90%, the variation of atomic contrast matches well with our experimental results. When the proportion is 50%, the ambiguous contrast becomes most significant, and its intensity center approaches the middle plane of two Mn layers. So this may lead to the erroneous interpretation that there is an extra atomic column at the high-temperature mirror plane^27^. Moreover, “overlapping effect” also occurs in vortex core region. Because of the complex spatial configuration of vortex cores, we can see two thirds of Y columns show ambiguous contrast periodically in the center regions (see supplementary section S7).

### Atomic structure at vortex core

**Figure 5:**
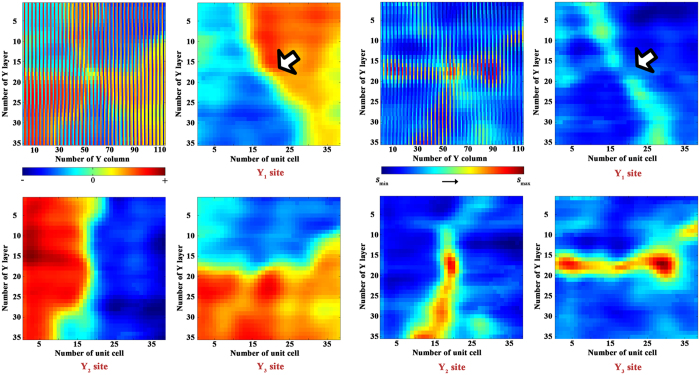
Atomic contrast analysis by means of 2D Gaussian fitting for the image of Fig. 4(a); mathematic fitting was used to map the variation of atomic contrast. (**a**) Distribution of displacement of the Y intensity center; the vortex structure is visible. The direction and magnitude of displacement is revealed by the color scale. The distributions of this value at three different crystallographic sites Y_1_, Y_2_, and Y_3_ are shown individually in (**b**–**d**), respectively. (**e**) Distribution of longitudinal standard deviation *s* which describes the degree of ambiguity of the contrast. The values for different sites are shown in (**f**–**h**). Coolness of color indicates clearer atomic contrast, while warmth of color corresponds to more ambiguous contrast. The white arrow indicates the region where the “overlapping effect” can be ignored near the vortex core.

In order to reveal the structural features at the vortex core, we used the two-dimensional (2D) Gaussian function to fit the intensity and position of each *R* atom in Fig. 4(a). After that, the contrast features can be described quantitatively by fitting parameters: the intensity centers are determined by the 2D peak positions and the degree of ambiguous is quantified by the longitudinal standard deviation (see supplementary section S8). Figure 5(a) is a map of displacement of the Y intensity center (determined by the deviation of each Y intensity center from the middle plane of two adjacent Mn layers: the value is negative (positive) when the intensity center is below (above) this plane), and Fig. 5(e) shows a map of the standard deviation, illustrating the elongation of the Y atomic image. These two mappings can be used to directly identify the change in position of atomic intensity centers and regions where “overlapping effect” exists.

Considering the triple superstructure in *R*MnO_3_, we can periodically label the *R* atoms as *R*_1_, *R*_2_ and *R*_3_ on the projective plane along the [110] direction (as shown in Fig. 3(a)). Figure 5(b–df–h) are maps of center deviation and longitudinal standard deviation for these three Y sites, respectively. In Fig. 5(b), the intensity center of the Y_1_ site shifts downward in the left area and upward in the right area, and these two areas transition smoothly over several unit cells. This means that the intensity centers shift upward gradually from left to right in this transition region. Similarly, the transition regions can also be found in Fig. 5c,d. Note that the standard deviation at the Y_1_ site falls to a low value in the vortex core region, as shown in Fig. 5(f), i.e. no “overlapping effect” exists here. So, the continuous change of intensity center shown in Fig. 5(b) exactly reflects the continuous atomic distortion at Y_1_ sites in core region. The continuous shift of Y atoms demonstrates that the atomic structure within the intersection of the six domains is differs from the domain regions and hence cannot be described by *P*6_3_*cm* symmetry. Instead, this structural feature coincides with the 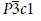 and *P*3*c*1 symmetry as analyzed in a previous section. Based on the span of this transitional area, the diameter of the core region can be estimated to be about 50 Å (see supplementary section S9 and Fig. S11). However, this value is much larger than the result given in ref. 32 (about 4 unit cells); this discrepancy may originate from the fact that, in that paper, the “overlapping effect” was not considered and the core region was determined simply by visual inspection instead of quantified measurement. As we discussed above, the real atomic positions may be misjudged due to the “overlapping effect”.

## Discussion

In summary, the structural features of domain wall and vortex core in YMnO_3_ have been investigated. The transformation of topological defects associated with symmetry breaking of the degeneracy space has been studied via homotopy group analysis, showing that the formation of “string-wall-bounded” structures is closely related to the shrinkage of the degeneracy space during cooling. Order parameter components within topological defects always deviate from those in a low-temperature degeneracy space, and this suggests that some energetically unfavorable structures corresponding to high-temperature *U*(1) continuous symmetry are preserved to maintain topological integrality of the vortex core. On the other hand, these microstructural features are directly revealed by our atomically resolved observations with consideration of the widespread “overlapping effect”, and the size of the vortex core region is estimated to be 50 angstroms in diameter. In these two aspects, our results provide some important clues for understanding the formation and proliferation of vortices in the *R*MnO_3_ system, and some new evidence for understanding the special structural features of the system’s characteristic topological defects, which could be important for exploring other topological defects and investigating associated novel physical phenomena in condensed matter.

## Methods

The YMnO_3_, ErMnO_3_ and Y_1−x_In_x_MnO_3_ (x = 0.1 ~ 0.25) single crystals used in this work were grown via flux method. The typical crystals are plate-like, a few millimeters wide and 20–50 μm thick. The crystal structure properties of these samples were firstly characterized by powder X-ray diffraction (XRD) in a Bruker D8 Discover diffractometer. The single-crystal samples of YMnO_3_/Y_1−x_In_x_MnO_3_ for scanning transmission electron microscopy (STEM) observations were prepared by polishing and then ion milling. Information about the morphology of the topological defects was acquired via dark-field imaging by JEM 2100F. The atomically resolved HAADF images were acquired by *C*s-corrected STEM using a JEM ARM 200 with atomic resolution of ~0.09 nm. All STEM images shown in this paper are taken along the [110] zone axis. The SEM images are acquired by Hitachi S-4800.

## Additional Information

**How to cite this article**: Li, J. *et al.* Homotopy-Theoretic Study & Atomic-Scale Observation of Vortex Domains in Hexagonal Manganites. *Sci. Rep.* **6**, 28047; doi: 10.1038/srep28047 (2016).

## Supplementary Material

Supplementary Movie 1

Supplementary Movie 2

Supplementary Movie 3

Supplementary Movie 4

Supplementary Movie 5

Supplementary Movie 6

Supplementary Information
