# Supplementary material for: Homotopy-Theoretic Study & Atomic-Scale Observation of Vortex Domains in Hexagonal Manganites
Source: Sci Rep. 2016 Jun 21;6:28047. doi: 10.1038/srep28047 (PMC4914935; doi:10.1038/srep28047)
Supplement: Supplementary Information [file srep28047-s7.pdf]

Homotopy-Theoretic Study and Atomic-Scale Observation of Vortex Domains in Hexagonal Manganites

Jun Li[1,6†], Fu-Kuo Chiang[1,5†], Zhen Chen[1,6], Chao Ma[1], Ming-Wen Chu[2], Cheng-Hsuan Chen[2,3], Huanfang Tian,[1] Huaixin Yang,[1,6] and Jianqi Li[1,4,6*]

[1]*Beijing National Laboratory for Condensed Matter Physics, Institute of Physics, Chinese Academy of Sciences, Beijing 100190, China*

[2]*Center for Condensed Matter Sciences, National Taiwan University, Taipei 106, Taiwan*

[3]*Department of Physics and Astronomy, Rutgers University, Piscataway, New Jersey 08854, USA*

[4]*Department of Physics and State Key Laboratory of Low-Dimensional Quantum Physics, Tsinghua University, Beijing 100084, China*

[5]*National Institute of Clean-and-Low-Carbon Energy, Beijing 100209, China*

[6]*University of Chinese Academy of Sciences, Beijing 100049, China*

†These authors contributed equally to this work.

*Correspondence and requests for materials should be addressed to J. Q. Li (email: ljq@iphy.ac.cn)

Supplemental Movies

Three movies shown here correspond to three simulated annealing processes with different annealing temperature (T_a). The initial states of simulations are set to be the same and only domain walls are included. This state is a low-energy state and can be stable at low temperature. Firstly, the temperature T increases gradually from 0 to 1200 K ($T_a < (T_s = 1270$ K)), 1250 K ($T_a \sim T_s$), and 1300 K ($T_a > T_s$), respectively in three systems. Then, an isothermal period (annealing process) is followed by cooling process down to $T = 0$ gradually. We consider the cooling processes of these three systems: in the first scenario, the symmetry of system remains to be Z_6 all the time, so no vortices can be observed in Movie I; in both the other two scenarios, the temperatures are high enough to drive the systems into continuous $U(1)$ symmetry, the cooling processes will inevitably experience a symmetry transition and the vortex cores formed at high temperature are preserved after the transition (as shown in Movie II and III).

Movie I | Simulated annealing process with T_a = 1200 K. Movie I – amplitude.mov and Movie I – phase.mov show the distribution of order parameter field Q and φ, respectively. No vortices appear because of no symmetry change of degeneracy order parameter space during this process.

Movie II | Simulated annealing process with T_a = 1250 K. Movie II – amplitude.mov and Movie II – phase.mov show the distribution of order parameter field Q and φ, respectively. The temperature is high enough to drive system into $U(1)$ symmetry. This provides the necessary condition for vortex core formation.

Movie III | Simulated annealing process with T_a = 1300 K. Movie III – amplitude.mov and Movie III – phase.mov show the distribution of order parameter field Q and φ, respectively. The temperature is high enough to drive system into $U(1)$ symmetry. This provides the necessary condition for vortex core formation.

Supplemental Information

Section S1. Landau free energy and degeneracy order parameter space of RMnO$_3$

For hexagonal manganites RMnO$_3$, S. Artyukhin *et al.* have given the free energy density expansion based on Landau theory [1]. It can be represented in the following form:

$$\begin{cases} f_{\text{tot}} = f_{\text{bulk}} + f_{\text{grad}} \\ f_{\text{bulk}} = \left(\frac{-a}{2T_s}(T - T_s)Q^2 + \frac{b}{4}Q^4 + \frac{c}{6}Q^6\right) + \left(\frac{g'}{2}Q^2P^2 + \frac{a_P}{2}P^2 + \frac{c'}{6}Q^6 \cos 6\varphi - gQ^3P\cos 3\varphi\right) \\ f_{\text{grad}} = \frac{1}{2}\sum_{i=x,y,z}\left[s_Q^i(\partial_i Q\partial_i Q + Q^2\partial_i\varphi\partial_i\varphi) + s_P^i\partial_i P\partial_i P\right] + \frac{1}{2}t_P^x\left[\left(\frac{\partial}{\partial x^2} + \frac{\partial}{\partial y^2}\right)P\right]^2 \end{cases} \quad (1)$$

where f_{bulk} is the bulk free energy density and f_{grad} is the gradient energy density. Since the primary order parameter of improper ferroelectric transition has at least two components, scalar variables Q and φ are used to describe the amplitude and azimuthal angle of trimerizing tilt, respectively. Spontaneous polarization is a secondary order parameter in this system, which is quantified by the amplitude (P) of polar Γ_2^- mode. It is triggered by the distortion of MnO_5^{3+} bipyramids. All coefficients in equation (1) had been determined by a series of first-principles calculations in Ref. 1. The coefficient of Q^2 varies with temperature T, $T_s \approx$ 1270K is the critical temperature of structural phase transition for YMnO$_3$. The bulk free energy density can be decomposed into two parts (separated by blankets in equation (1)), denoted as f_1 and f_2, respectively, where f_2 breaks the full symmetry of f_1.

Considering a crystal in mono-domain state, the equilibrium state of order parameters can be determined by minimization of f_{bulk} with respect to Q, φ and P. This gives Q_0 = 0.956 Å (when T = 0 K), φ belongs to Φ = [0, π/3, 2π/3, π, 4π/3 and 5π/3], and $P = gQ^3\cos 3\varphi/(a_P + g'Q)$. Hence, the order parameter space $\boldsymbol{R}_1$ of this uniform system is six evenly spaced points on the edge of a circle. As the temperature rises, both the bulk free energy density and relative weight of f_1 in f_{bulk} increases, as shown is Fig. S1. When temperature approaches T_s,

f_{bulk} varies little with different φ and f_1 becomes dominant in f_{bulk} (i.e. $|f_1| >> |f_2|$), the minimization of f_{bulk} gives decreased Q value and depends little on the variation of φ. Then the degeneracy space expands from $\boldsymbol{R_1}$ to $\boldsymbol{R_2}$. When $T > T_s$, $-a(T - T_s)/2T_s$ becomes positive, and $Q = 0$ gives the minimum of f_{bulk}. Then the circle $\boldsymbol{R_2}$ shrinks into a single point $\boldsymbol{R_3}$. Above all, the degeneracy order parameter space is a function of temperature.

Section S2. Homotopy groups and exact homotopy sequences

According to homotopy theory, topological defects can be classified by the elements of homotopy groups associated with the symmetry of order parameter space. The zeroth homotopy group $\pi_0(\boldsymbol{R}, x_0)$ classifies two dimensional domain walls (x_0 is a base point in order parameter space $\boldsymbol{R}$), the fundamental homotopy group $\pi_1(\boldsymbol{R}, x_0)$ classifies one dimensional vortex lines, and the second homotopy group $\pi_2(\boldsymbol{R}, x_0)$ classifies the point defects. In these groups, x_0 is a base point in order parameter space $\boldsymbol{R}$ and it can be omitted when $\boldsymbol{R}$ is a connected space. These groups are the special cases of the so-called relative homotopy groups. If A is a subset of $\boldsymbol{R}$ which contains the base point x_0, the n*th* relative homotopy group can be expressed as $\pi_n(\boldsymbol{R}, \boldsymbol{A}, x_0)$. When the degeneracy parameter is restricted to $\boldsymbol{A}$, for instance on surfaces, or in regions of small parameter variations, we can use relative homotopy groups to classify these topological defect structures. In addition, relative homotopy groups have the following relationship:

$$\ldots \rightarrow \pi_{n+1}(\boldsymbol{R},\boldsymbol{A},x_0) \xrightarrow{i} \pi_n(\boldsymbol{A},x_0) \xrightarrow{j} \pi_n(\boldsymbol{R},x_0) \rightarrow \pi_n(\boldsymbol{R},\boldsymbol{A},x_0) \ldots$$
$$\ldots \pi_2(\boldsymbol{R},\boldsymbol{A},x_0) \rightarrow \pi_1(\boldsymbol{A},x_0) \rightarrow \pi_1(\boldsymbol{R},x_0) \rightarrow \pi_1(\boldsymbol{R},\boldsymbol{A},x_0) \rightarrow \pi_0(\boldsymbol{A}) \rightarrow \pi_0(\boldsymbol{R}),$$

in which "→" represents homomorphic mapping, and this series of mappings is called exact homotopy sequence. In this sequence, the image (im) of one homomorphism always equals to the kernel (ker) of the next homomorphism, i.e.

$$\text{im}\ (i) = \text{ker}\ (j).$$

For RMnO$_3$, our experimental results demonstrated that only domain walls can appear as topological defects when the degeneracy space of a system remains at $\boldsymbol{R_1}$ even temperature varies, i.e. only stripe, circle and annular domains can be obtained in ErMnO$_3$ after an annealing process below T_s, as shown in Figs. S2(a-c). A crystal with straight condensed stripe domains is chosen to be the original state. All of these kinds of domains are essentially the same though with different shapes. From the perspective of topology, they are all domain walls. So this result matches well with the theoretical description. In addition, the thermal process in which $\boldsymbol{R_2}$ becomes dominant is a necessary condition for the appearance of vortices. As shown in Fig. S2(d), after an annealing process above T_s, the coexistence state of vortices ("string-wall" bounded type) and circle domains can be observed.

Section S3. Evolution of order parameter field with temperature and external electric field

To consolidate the result obtained from homotopy theory analysis, the evolution of order parameter field with temperature is simulated on a two dimensional rhombic lattice with periodic boundary condition, based on the Landau phenomenological model (see equation 1). Each site of the lattice is assigned two variables Q and φ whose values are updated by the Metropolis algorithm. Three typical snapshots of the evolution are shown in Figs. 2(a-c), corresponding to three states at $T > T_s$, $T_s > T >> 0$ and $T = 0$, respectively. Figure 2(a) shows a defect-free homogenous state with all Q values in close proximity to 0. Just below the phase transition temperature, Q increases slightly in the demand of minimization of bulk free energy which is still non-sensitive to the value of φ, so there is no preference for φ. However, the spatially configurations of order parameter field has noticeable impact on the gradient energy, so smooth variation of φ among adjacent sites is energetically preferred. The system in this state is in analogy with the low-temperature phase of the *x*-*y* model in spin system because their degeneracy spaces both adopt $U(1)$ symmetry, then topological excitations (bounded vortex-antivortex pairs without domain walls) will appear to minimize the total energy, as shown in Fig. 2(b). Further decrease of temperature result in much larger value of Q, and this drives the system into six-fold degeneracy state to minimize the bulk free energy. Domain/domain wall emerges for balancing the local bulk energy and gradient energy, and then as shown in Fig. 2(c), each core is surrounded by six domain walls. It is noticeable that the position of vortex cores changes little from Fig. 2(b) to Fig. 2(c) and no nucleation or annihilate of cores can be observed, this implies that these strings are stable span a large temperature region.

The local configurations of domain wall and vortex core are determined by balance among f_1, f_2 and f_{grad}. In order to reduce gradient energy, some new order parameters which beyond the degenerated space may appear in spite of the additional cost in bulk free energy. Close to the wall, the increase of gradient energy density results in deviation of Q and φ from $\boldsymbol{R}_1$. The value of φ is no longer confined to those six distinct values as in the region away from defects. Instead, it spreads into continuous distribution. Supposing that φ changes sharply between two adjacent domains, such as from 0 to π/3 between α^+ and β^-, then the gradient energy diverges. In order to remove this singular gradient, φ has to vary from 0 to π/3 continuously in the order parameter field even though this area will be narrow. This is coincided with the first principle calculation results showed in Ref. 2. Because of the positive coefficient of the term which contains Q^2 in f_{grad}, decrease of Q within domain wall leads to lower gradient energy. The value range of φ is associated with the value of Q. When Q diverges slightly from Q_0, possible values of φ extend to the neighborhood of six values in Φ. These six discrete components do not connect each other until Q drops to a critical point. This value can be determined by minimization of f_1 which gives $Q_1 = 0.871$ Å at $T = 0$K. So, the minimum value of Q in each wall region should be smaller than Q_1. The width of wall and minimum value of Q are associated with the magnitude of stiffness parameters in f_{grad}. From this point of view, there should be no essential difference between those two kinds of domain walls in $R\text{MnO}_3$ (APB1+FEB and APB2+FEB, APB: antiphase boundary, FEB: ferroelectric boundary). So the answer to the question "whether there is an extra atomic column stays at the high temperature mirror plane" should be the same for APB1+FEB and APB2+FEB. Because of higher gradient energy within vortex cores, more evident structural deformation (atomic distortion) is expected to be

observed in this region.

Having obtained the distribution of Q and φ, it is convenient to calculate the configuration of secondary order parameter P near vortex core by using equation $P = gQ^3 \cos 3\varphi/(a_P + g'Q)$. The result is shown in Fig. S3(a), P changes sign in adjacent domains and varies to nearly zero inside vortex cores. Since P is proportional to the spontaneous polarization, the distribution of polarization can also be reflected from this figure. The polarization within a vortex core is obviously weaker than domain regions, so the stability of core should be less sensitive to external electric field. We also performed numeric simulations by introducing a static electric energy $f_E = -E \cdot P$, domain pattern shown in Fig. 2(c) is chosen to be the original state for Monte Carlo simulation. Figures S3(b) and (c) show the effect of two opposite electric field. The position of domain walls changes dramatically in both figures. The polarization direction of large-area domains is parallel to the applied electric field. Domains with the same φ value can coalesce together, while domains with $|\Delta\varphi| = 2\pi/3$ are always separated by two domain walls (a narrow domain). It is notable that the position of vortex cores changes little under electric field. This high stability of vortex cores has been verified by previous reports and our own experiments.

Related experimental results are shown in Fig. S4. Figure S4(a) presents the schematic of experimental setup: the gray layers are silver electrodes and samples are soaked in silicone oil to avoid high-voltage discharge. The applied field is E = 750 kV/cm, which is much higher than previous reported works. When the original state is stripe and annular domain pattern in $ErMnO_3$, external electric field reverses the polarization of large-area domains, and annular domains can still be observed under electrodes, as shown in Fig. S4(b). Bright lines shown in Fig. S4(c) represent domain walls in $YMnO_3$, the blurry contrast results from re-distribution of charge carries driven by electric field. This secondary electron image shows the domain pattern after field applied. Figure S4(d) is a back-scattered electron image which shows the surface topography etched by acid, it reflects the original domain pattern before field applied. By comparing these two images, we found that the position of vortex cores is still stable under such high field, as indicated by arrows shown in Fig. S4(c) and Fig. S4(d).

Section S4. Relationship between order parameters and structural distortion

In the above analysis, we consider the RMnO$_3$ crystal as a continuous medium which is characterized by the order parameter field. In practice, the variation of order parameters can reflects the atomic distortion of crystal lattice. According to group theory, the condensation of the K_3 mode lowers the symmetry from $P6_3/mmc$ to $P6_3cm$. Q reflects the relative strength of K_3 mode and its value is determined by the atomic displacement driven by condensation of K_3 mode, i.e.

$$Q = \sqrt{2(v_{K_3}^{\mathrm{Y1}})^2 + 4(v_{K_3}^{\mathrm{Y1}})^2 + 6(v_{K_3}^{\mathrm{Mn}})^2 + 6(v_{K_3}^{\mathrm{O1}})^2 + 6(v_{K_3}^{\mathrm{O2}})^2 + 2(v_{K_3}^{\mathrm{O3}})^2 + 4(v_{K_3}^{\mathrm{O4}})^2}$$

Normally, the displacement value at each site decreases with smaller Q, so the distortion in R layers and tilting of MnO_5^{3+} bipyramids changes accordingly with Q. Group theoretical analysis shows that there are three isotropy subgroups $P6_3cm$, $P\bar{3}c1$ and $P3c1$ for the K_3 irreducible representation of space group $P6_3/mmc$, so these three kinds of structures are symmetry-permitted to exist in RMnO$_3$ system, see Table S1. It is known that when $\varphi \in \Phi$, the space group is $P6_3cm$ and R layers adopt the "up-up-down" or "down-down-up" configuration (Fig. S5(a)). For space group $P\bar{3}c1$, the azimuthal angle φ of MnO_5^{3+} bipyramids belongs to the set $\Phi' = \{\pi/6, \pi/2, 5\pi/6, 7\pi/6, 3\pi/2$ and $11\pi/6\}$. In such unit cell, one third of the R atoms distort upward, one third of them distort downward and the rest keep in position, as shown in Fig. S5(b). This configuration is similar to the centrosymmetric hexagonal $InMnO_3$ structure. In contrast, $P3c1$ imposes the least symmetry restrictions on the atomic coordinates. So when $\varphi \notin (\Phi \cup \Phi')$, the symmetry can be described by $P3c1$ and R atoms have three different Wyckoff positions (2(a), 2(b) and 2(c)). The direction and magnitude of R displacements along the $\boldsymbol{c}$ axis at each Wyckoff position are not restricted by any symmetry element, so the vertical coordinate z of R_1, R_2 and R_3 (at 2(a), 2(b) and 2(c), respectively) can be different to each other on the same layer of a unit cell, as shown in Fig. S5(c). So, these three kinds of structures can be distinguished by the distortion state of R layers. Since the structure phase transitions are induced by the size mismatch between R and Mn, different tilt state of bipyramids should be accompanied by different R buckling state. As φ varies from 0 to 2π, phase $P6_3cm$ and phase $P\bar{3}c1$ appear alternately. Phase $P3c1$ appears as an intermediate state between these two phases. This relationship between displacement Δz of R atom at each Wyckoff position and φ is shown schematically in Fig. 3(b).

In order to verify this relationship, we carried out first-principles calculations on $YMnO_3$ using the projector-augmented wave method as implemented in Vienna Ab initio simulation package (VSAP). We used the LSDA+U method to describe the exchange-correlation functional with values of $U = 8$ eV and $J = 0.88$ eV that were the same as previous reports. The ground state structure was adopted from x-ray diffraction experiments and A-type magnetic configurations were adopted. All atoms were fully relaxed until the forces converged to 0.005 eV ·Å. We constructed a structural model by rotating the tilting angle of the MnO_5^{3+} bipyramids by $\pi/6$ and performed the structural relaxations with the positions of the apical oxygen atoms fixed and all other atoms relaxed. After the relaxation, we found that the configuration of Y atoms changes from "up-down-down" to "up-middle-down". Though the middle Y atom is not accurately in the center of the nearest two Y atoms in the plane (may be induced by the fixed Mn-O_{ap} bond length), a significant shift along the c axis occurs. As shown in Fig. S5(a), Y_1 shifts down 0.0582 Å and Y_3 shifts down 0.0125 Å which are obvious smaller compared to the upward 0.1141 Å shift of Y_2, while the changes of lattice constants are negligible. The total energy of this unstable structural state is 0.34 eV higher than that of the fully relaxed ground state. Therefore, when the tilt state of MnO_5^{3+} bipyramids changes from the $P6_3cm$ structure, the Y atoms will move towards their paraelectric positions accordingly and the transitional structures belong to $P\bar{3}c1$ or $P3c1$ owning to the symmetry requirements.

Based on the above discussions, we can safely conclude that the displacement Δz of each R atom along the $\boldsymbol{c}$ axis is a function of variables Q and φ. Since the energy of phase $P\bar{3}c1$ and $P3c1$ are relatively higher than $P6_3cm$, these two structures can just exist in the regions where the strain accumulates, i.e. where the distribution of order parameters are not uniform. The results of first-principles calculations presented in Ref. 2 also indicate that φ deviates a little from Φ within several unit cells near domain walls. However, the impact of non-uniform order parameter field on the atomic structure should be more significant in the core regions because of higher gradient energy density. Continuous variation of φ is accompanied by remarkable decrease of Q around the cores, so the atomic structure of vortex core is more likely to be constructed by $P\bar{3}c1$ and $P3c1$, and the buckling state of R layers may present some differences from the normal $P6_3cm$ phase.

Section S5. Atomic structure of vortex core observed by STEM

Our experimental results have showed that the light substitution of In for Y could visibly increase the density of vortex cores, which is technically important for high-resolution TEM observations to image a vortex core within an area as small as a micron [3]. A typical atomic-resolved HAADF image of vortex core is shown is Fig. S6.

As described in Ref. 4, $P\bar{3}c1$ phase is the ground-state structure for hexagonal $InMnO_3$. In contrast, $P\bar{3}c1$ phase in $YMnO_3$ is a high energy phase which can only exist within topological defects. Therefore, to our knowledge, the doped In atoms may be distributed in vortex core regions for releasing the stress there. The existence of In atoms may lower the energy cost for vortex core formation. This might be the reason why we have higher vortex core density in In doped sample. However, considering the low level of doping (10%), the general atomic configurations of topological defects should not be affected by doped In atoms. Because of the local energy relaxation from In atoms, the measured core size in $Y_{0.9}In_{0.1}MnO_3$ might be a little smaller than in $YMnO_3$.

Section S6. Three-dimensional structure of ferroelectric domain in *R*MnO₃

The three-dimensional character of defects is an essential factor to consider in analyzing the two-dimensional projected HAADF images. To visualize the distribution of vortex strings and domain walls, a 3D numerical simulation is performed on a 200×200×50 lattice with periodic boundary condition. The result obtained after a simulated annealing process is shown in Fig. S8(a). From the distribution of φ and Q, vortex-antivortex pairs can be observed on any surface, this is consistent with the experimental observation as shown in Fig. S8(d). In order to visualize the distribution of defects alone, the stereostructure of a single domain and vortex strings are shown in Fig. S8(b) and (c), respectively. Different from the plate-like domain walls in $BaTiO_3$ or other proper ferroelectrics, the domain walls in RMnO$_3$ appear as curved surfaces, terminating at the

surfaces or vortex strings. Two reasons may result in this difference: firstly, the annihilation of vortex-antivortex pairs is an "astronomical" time scale process, so these curved walls are topologically protected by the vortices from high temperature; secondly, the $YMnO_3$ is a kind of semiconductor-like ferroelectric with relative high density of carriers, so the unfavorable electrostatic at the charged walls can be lowered by the directional movement of carriers. Similar to other string-type topological defects, such as disclinations, they form loops inside the medium or terminate on the surfaces. According to these features, we cannot expect that the walls are always parallel to the observation directions. Actually, the bend of domain wall can happen at the atomic level [2], and it is estimated that there are about 50 atoms in each column along the observation direction even within a 30 nm thick sample, so the overlap of domains is nearly inevitable.

Section S7. The "overlapping effect" near vortex core

We construct a simplified three-dimensional atomic model which contains sharp domain walls and straight vortex string to analyze the impact of "overlapping effect" on the atomic contrast as shown in Fig. S9. Because the domain walls diverge radially from the vortex core with 60°-angle between neighboring domain walls as described in Ref. 5, there are two domain walls are set to be parallel to the [110] direction in the vicinity of core in our model. In such situation, there are three domains overlap along the observation direction at most. Two thirds of Y columns contain the oppositely distorted atoms in the region where three domains overlap, as shown in Fig. S10(a), so the contrast at these sites should be diffused in experimental image. The projection of our atomic model along the [110] direction is shown in Fig. S10(b). The light green parts are pure domains corresponding to α^+, β^-, γ^+, α^-, β^+ and γ^-; the light yellow parts correspond to the projections of individual inclined domain walls and the light blue part is exactly the integration of three domains (left: γ^- - β^+ - α^-, right: α^+ - β^- - γ^+). The yellow circles, red circles and rhombus represent the upward, downward and diffused Y atoms, respectively. According to this schematic, domains are separated by belts in which one third of Y atoms should show diffused contrast, while only one third of Y atoms show explicit contrast in the vicinity of vortex core. The size, shape and detailed structure of these special regions are associated with the 3D structure of vortex strings and domain walls and the thickness of crystal. Such a complicated pattern is generated from a simplified model in which the vortex core on the ***ab*** plane is assumed to be a single point. It is arbitrary to determine the atomic structure of vortex core immediately from the experimental images without careful contrast analysis, because not all the intensity centers reflect the accurate atomic positions, especially in regions with high defect density. In order to acquire more valuable information, we have to process the explicit atomic contrast in detail to illuminate the real migrations of atom.

Section S8. Two-dimensional Gaussian fitting

Firstly, the image shown in Fig. 4(a) is divided into many conterminous rectangle pieces. In each piece, just one atom image is contained and the edges are defined by the minimum of local intensity. Then, the intensity distribution in each piece is fitted by function:

$$f(x,y) = Ae^{-\left[\frac{(x-x_0)^2}{2\sigma_x^2}+\frac{(y-y_0)^2}{2\sigma_y^2}\right]}.$$

The intensity centers can be determined by the fitting parameter (x_0, y_0) and the degree of ambiguous can be quantified by the longitudinal standard deviation σ_y.

In Fig. 5(a), we define the displacement of Y column intensity center as the deviation from the averaged center line between two adjacent Mn layers. It is notable that the Mn layers also present ups and downs configuration, this may origins from the contrast of O ions which is very close to the Mn ions on the projective plane. As shown in Fig. 4(e), though the positions of Mn ions are set to be at the same level in structural model, the fluctuation can still be observed. For this reason, the averaged position of Mn ions in each layer is calculated and used as reference lines. The values are shown as negative (positive) for upward (downward) poling in Fig. 5(a), then the cool tone dominates in the downward polarized domains (α^-, β^- and γ^-) where two thirds of *R* atoms shift downward in these regions; on the other hand, the other three upward polarized domains (α^+, β^+ and γ^+) are shown as the warm-toned regions.

Section S9. Measurement of vortex core size

To provide a more intuitive result, we pick a region which cross the green box shown in Fig. 4(a), and a line scan is carried out for a single Y layer as shown in Fig. S11(a). The standard deviation and relative displacement along the ***c*** axis of Y_1, Y_2 and Y_3 are plotted in Fig. S11(b) and (c), respectively. Since the standard deviation of atom Y_1 stays at a low level, no significant elongated contrast appear even across the core region. Meanwhile, the contrast at Y_2 site only becomes ambiguous in the vicinity of center and its contrast recovers with increasing distance; atom Y_3 shows ambiguous contrast all over within this region. The clear contrast at the Y_1 site shows that these atoms shift upward gradually from the lower site to a higher site by spanning over about 10 unit cells. Then the size of vortex core is estimated to be 50 Å.

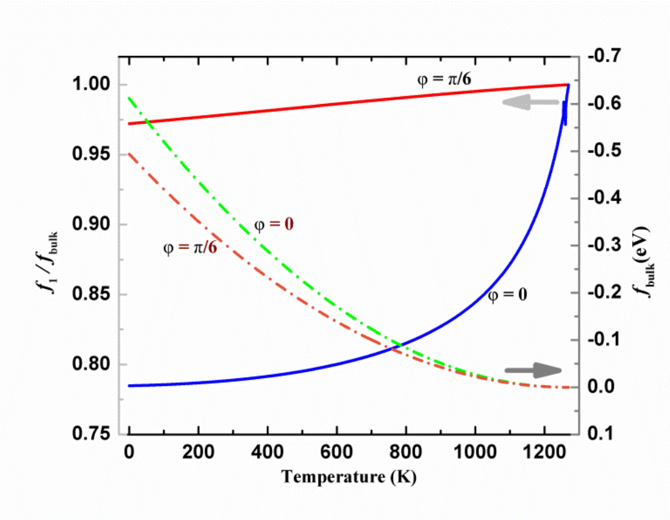

Fig. S1. Variation of f_1/f_{bulk} (solid lines) and f_{bulk} (dotted lines) with temperature when φ is fixed to be 0 and π/6, respectively. With rising temperature, the ratio f_1/f_{bulk} keeps increasing except some oscillations near T_s, this reveals that the magnitude of f_1 is always larger than f_2, especially just below T_s. The bulk free energy density f_{bulk} also increases with temperature, and it becomes almost φ independent at high temperature region.

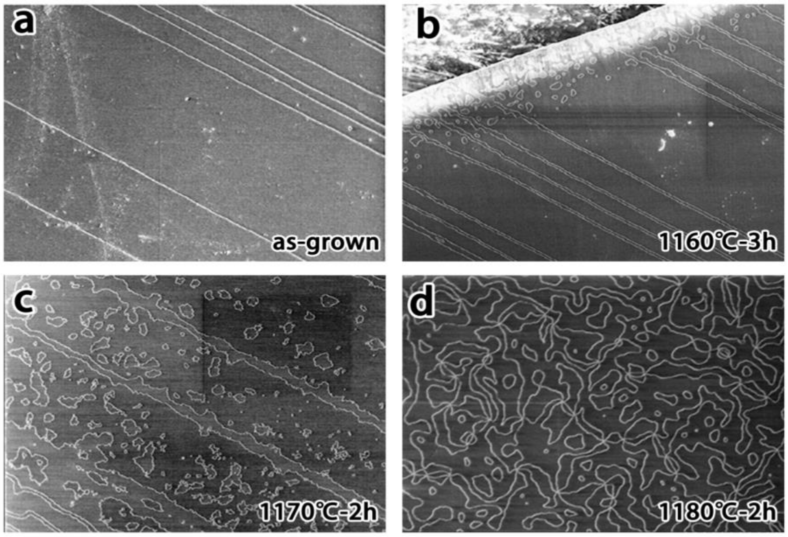

Fig. S2. Ferroelectric domains observed in $ErMnO_3$ via scanning electron microscope after different annealing conditions. Bright lines represent the position of domain walls. (a) Condensed stripe domains are considered as the original state for annealing. (b) The domain pattern observed after an annealing process at 1160 ˚C for 3 hours. (c) The sample is annealed at 1170 ˚C for 2 hours. (d) When the annealing temperature increases to 1180 ˚C, vortices appear after annealing.

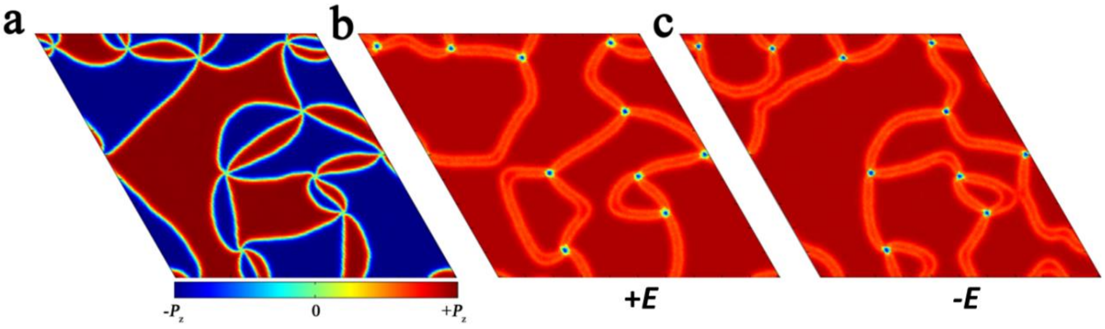

Fig. S3. (a) The distribution of secondary order parameter field *P*. (b) and (c) are simulated patterns obtained after an electric poling process, the electric filed is +*E* and –*E* respectively.

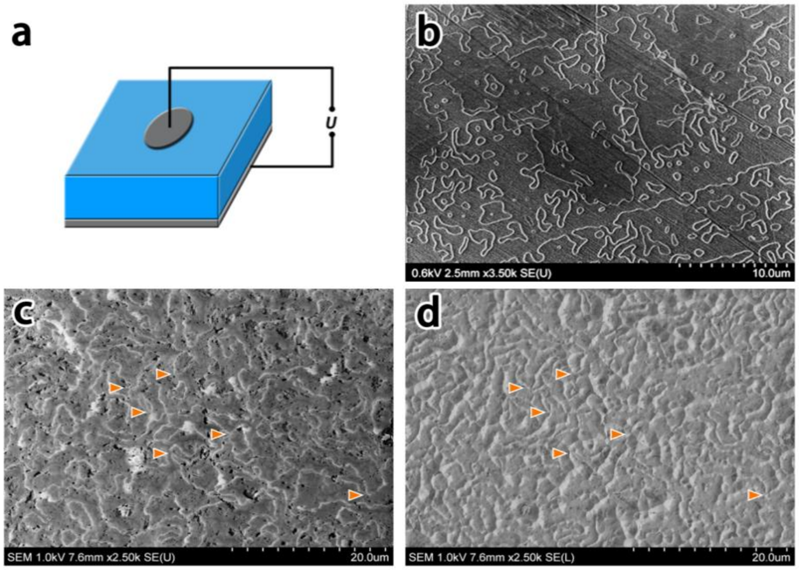

Fig. S4. (a) Schematic of experimental setup. (b) Domain pattern observed in $ErMnO_3$ after electric field applied. (c) and (d) are domain patterns observed in $YMnO_3$ after and before electric field applied, respectively.

*show direction vector
*display isotropy

Irrep (ML)	Subgroup	Dir		Basis Vectors	Origin
K3	185 P6_3cm	P1	(a,0)	(2,1,0),(-1,1,0),(0,0,1)	(0,0,0)
K3	165 P-3c1	P2	(0,a)	(2,1,0),(-1,1,0),(0,0,1)	(0,0,0)
K3	158 P3c1	C1	(a,b)	(2,1,0),(-1,1,0),(0,0,1)	(0,0,0)

Tab. S1. Three isotropy subgroups for the K_3 irreducible representation of space group $P6_3/mmc$.

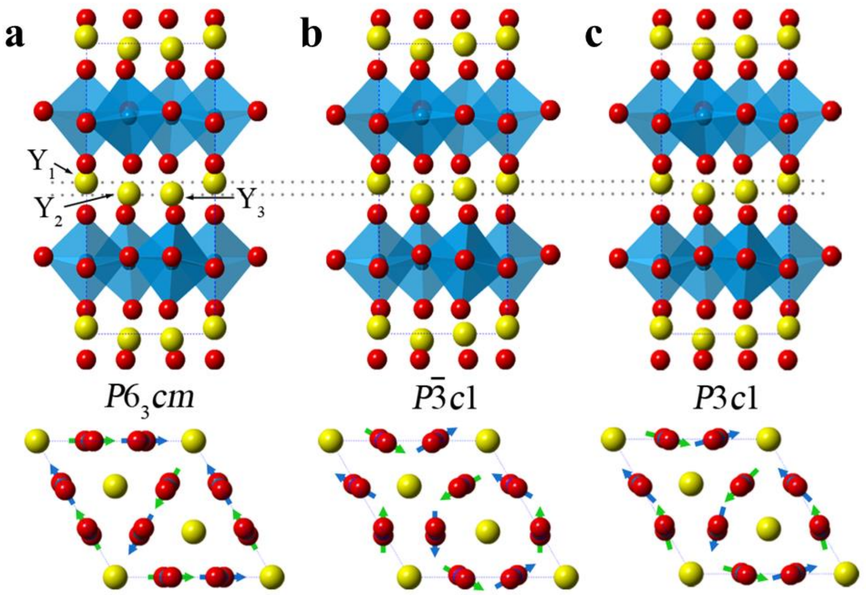

Fig. S5. (a) The RMnO$_3$ unit cell with $P6_3cm$ symmetry observed along the [110] (left) and [001] (right) directions. When this structure is distorted into $P\bar{3}c1$ or $P3c1$, the buckling state of R layers and the tilt direction of MnO$_5$ bipyramids will change accordingly, as shown in (b) and (c), respectively.

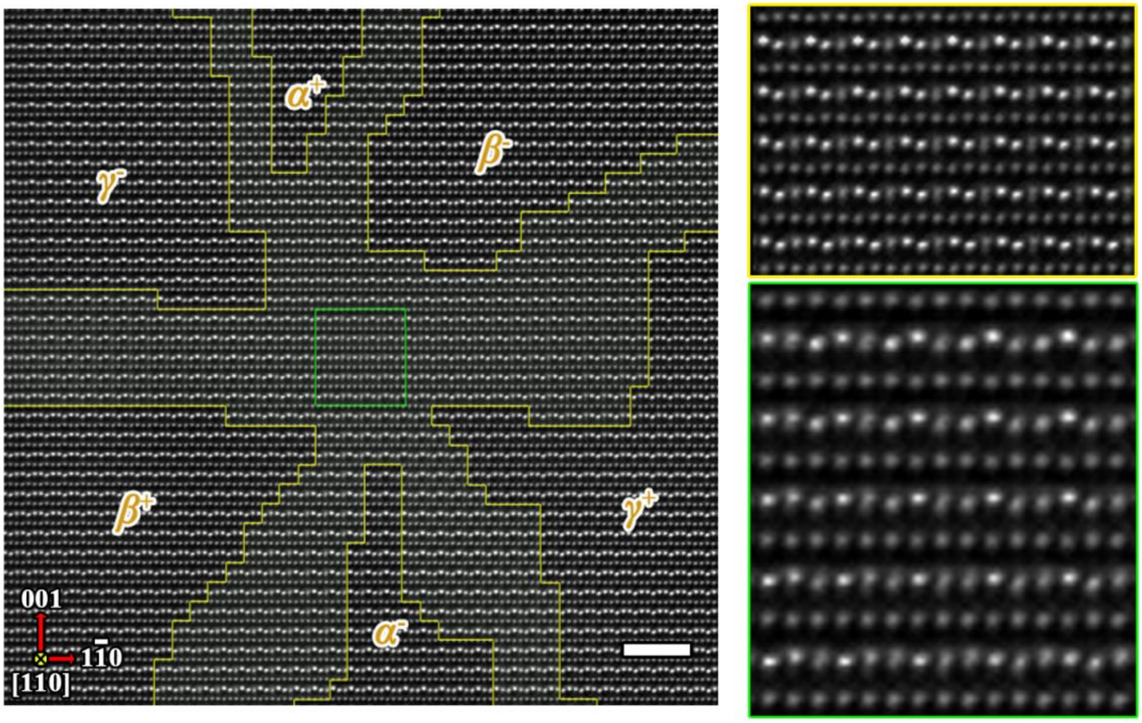

Fig. S6. A STEM image obtained in $Y_{0.9}In_{0.1}MnO_3$. Six kinds of domains appear alternately around the vortex core. The region in which the contrast of 1/3 Y atoms are ambiguous is delineated by yellow lines and the region in which 1/3 Y atoms are ambiguous is indicated by the green box. The typical contrast features of these two regions are shown in the enlarged boxes, respectively. Scale bar: 2 nm.

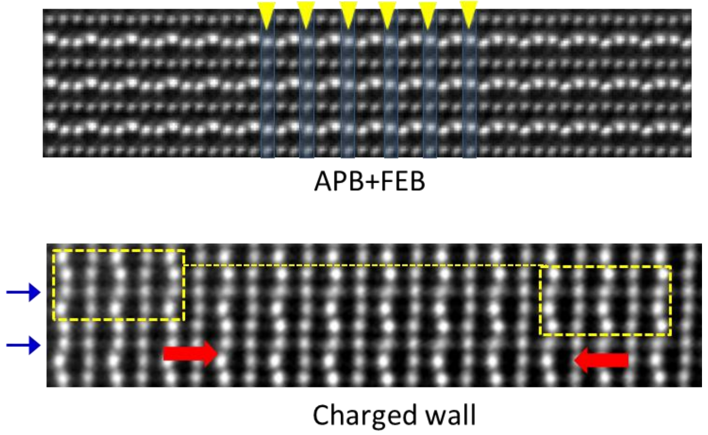

Fig. S7. “Overlapping effect” observed in other two kinds of domain walls. The upper figure shows the atomic contrast anomalies observed in a “side-by-side” type domain wall. The structural phase shift between two adjacent domains is different from the one shown in Fig. 4(b). The lower figure shows similar phenomenon observed in a “head-to-head” type domain wall.

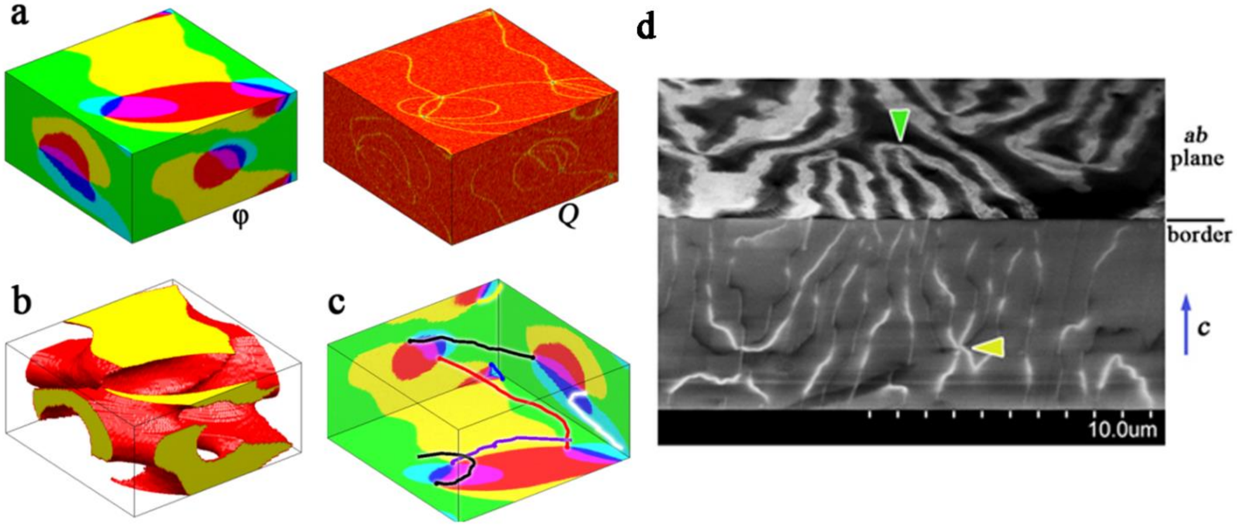

Fig. S8. (a) The simulated distribution of φ (left) and Q (right) field on a 200×200×50 3D lattice. (b) shows the stereostructure of β domain in (a), the domain wall presents as red curved surface. (c) The colored lines represent the position of vortex strings in 3D space. (d) shows the complicated 3D domain structure observed near the common border of two mutually perpendicular surfaces in $YMnO_3$.

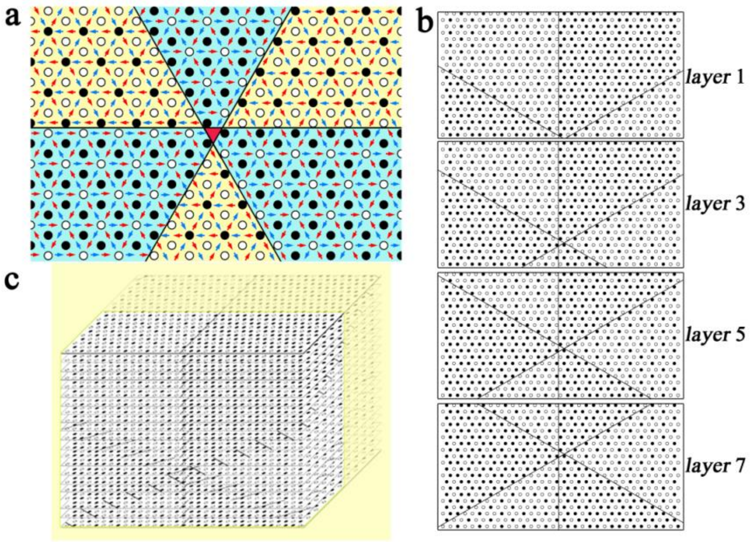

Fig. S9. (a) Simplified two dimensional atomic model for vortex structure, the vortex core is considered to be a single point (red region). Y ions are shown as bright and dark solid circles and the tilting direction of Mn-O bipyramids are indicated by arrows (the color indicates upper and lower Mn layers). In this model, the tilting direction within the core region is frustrated. (b) To construct a 3D model, the atomic structures of different layers are selectively shown. The vortex core just shift along one certain direction. (c) The projection along [110] direction can be obtained from this model.

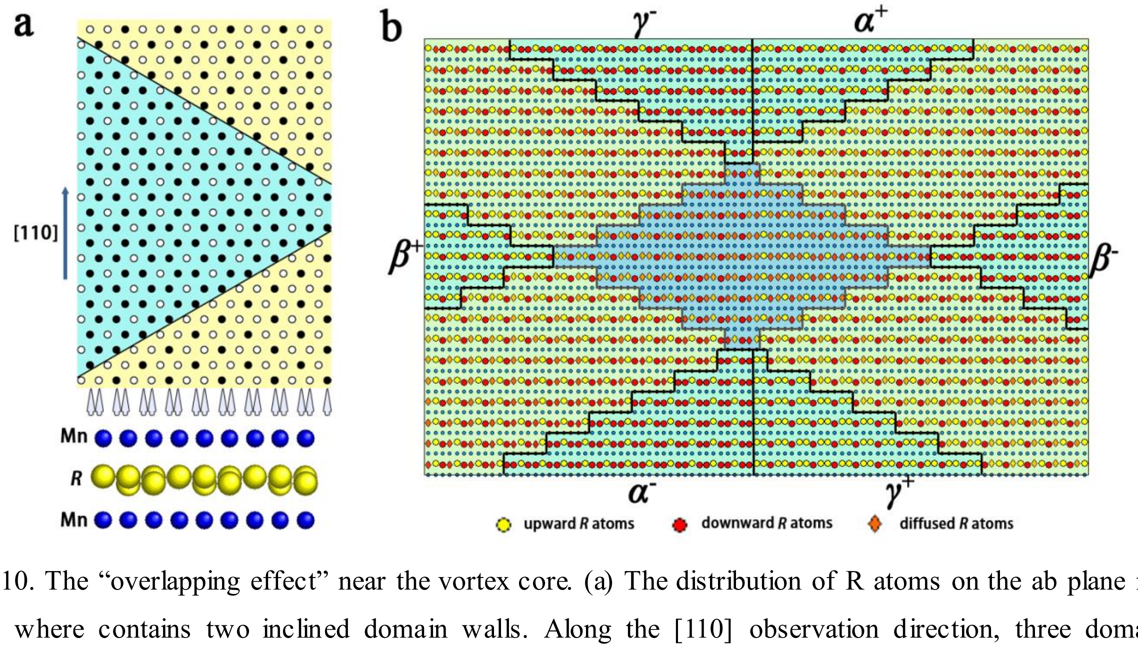

Fig. S10. The "overlapping effect" near the vortex core. (a) The distribution of R atoms on the ab plane in a region where contains two inclined domain walls. Along the [110] observation direction, three domains overlap, and the projection is shown in the lower panel. (b) Even suppose that the vortex core is a single point at which six domain walls meet on the ab plane, the stereo projection along the [110] axis also gives rise to complicated contrast features near the core region.

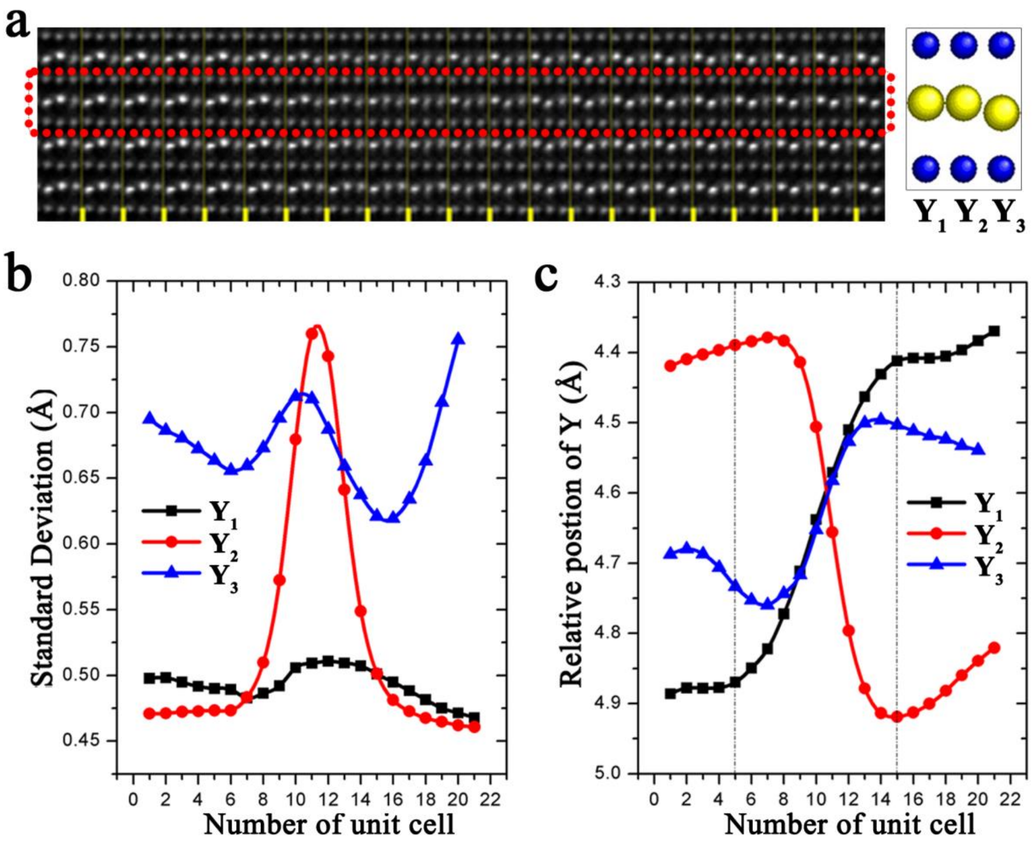

FIG. S11. (a) An enlarged region taken near the green box in Fig. 4(a). In each layer, we label the Y atoms as Y_1, Y_2, and Y_3 periodically from left to right. In order to analysis the atomic contrast in the red box labeled in (a), we fitted this Y layer by two-dimensional Gaussian function as shown in (b) for the variation of standard deviations and (c) for the shift of intensity centers.

References

1. S. Artyukhin, K. T. Delaney, N. A. Spaldin, and M. Mostovoy, Nat. Mater. **13**, 42 (2013).
2. Y. Kumagai and N. A. Spaldin, Nat. Commun. **4**, 1540 (2013).
3. L. Wang *et al.*, Chin. Sci. Bull. **59** (36), 5194 (2014).
4. Y. Kumagai *et al.* Phys. Rev. B 85, 174422 (2012).
5. S. C. Chae *et al.*, Phys. Rev. Lett. **110**, 167601 (2013).
